# Accuracy of freely available online GFR calculators using the CKD-EPI equation

**DOI:** 10.1007/s00228-020-02932-x

**Published:** 2020-06-19

**Authors:** Sarah Seiberth, Theresa Terstegen, Dorothea Strobach, David Czock

**Affiliations:** 1 Hospital Pharmacy, University Hospital, LMU Munich, Munich, Germany; 2 Doctoral Program Clinical Pharmacy, University Hospital, LMU Munich, Munich, Germany; 3 Department of Clinical Pharmacology and Pharmacoepidemiology, University of Heidelberg, Im Neuenheimer Feld 410, 69120 Heidelberg, Germany

**Keywords:** Estimated glomerular filtration rate, CKD-EPI equation, Online calculator, Drug dose adjustment

## Abstract

**Purpose:**

Estimated glomerular filtration rate (eGFR) as calculated by the Chronic Kidney Disease Epidemiology Collaboration (CKD-EPI) equation is used for detection of chronic kidney disease and drug dose adjustment. The purpose of the present study was to investigate the accuracy of freely available eGFR online calculators.

**Methods:**

All identified CKD-EPI online calculators were run with five reference cases differing in age, sex, serum creatinine, and ethnicity. Conversion from eGFR_indexed_ (unit ml/min per 1.73 m^2^) to eGFR_non-indexed_ (unit ml/min) and creatinine unit from milligramme/decilitre to micromole/litre was checked, if available.

**Results:**

Only 36 of 47 calculators (76.6%) produced accurate eGFR results for all reference cases. Eight of 47 (17.0%) calculators were considered as faulty because of errors relating to ethnicity (4 calculators), to conversion of the eGFR unit (2 calculators), to erroneous eGFR values without obvious explanation (2 calculators), to conversion of the creatinine unit (1 calculator), and to an error in the eGFR unit displayed (1 calculator). Overall, 28 errors were found (range 59 to 147% of the correct eGFR value), the majority concerning calculation of eGFR_indexed_ and the conversion to eGFR_non-indexed_. Only 7 of 47 (14.9%) calculators offered conversion of the eGFR unit.

**Conclusions:**

Erroneous calculations that might lead to inappropriate clinical decision-making were found in 8 of 47 calculators. Thus, online calculators should be evaluated more thoroughly after implementation. Conversion of eGFR units that might be needed for drug dose adjustments should be implemented more often.

**Electronic supplementary material:**

The online version of this article (10.1007/s00228-020-02932-x) contains supplementary material, which is available to authorized users.

## Introduction

In clinical routine, glomerular filtration rate (GFR) is most commonly estimated with the creatinine-based Chronic Kidney Disease Epidemiology Collaboration (CKD-EPI) equation, which is used for detection and staging of chronic kidney disease (CKD) [1, 2]. Drug dose adjustments in patients with renal impairment are traditionally based on creatinine clearance, using either the endogenous creatinine clearance or, more easily, Cockcroft and Gault’s equation [3, 4]. In this regard, creatinine clearance estimates should not be generally replaced by GFR estimates [5]. In elderly patients, for example, higher estimates by GFR equations could lead to inappropriately high doses [6–8] and an increased risk of adverse events, e.g. bleeding risk in case of anticoagulants [9, 10]. Nevertheless, the GFR is already used for dose adjustments (e.g. carboplatin) and for contraindications (e.g. metformin) of some drugs and GFR equations will presumably be used increasingly also for drug dose adjustment in clinical routine and for approval of new drugs [11].

The creatinine-based CKD-EPI equation requires age, sex, ethnicity, and a standardised measurement of serum creatinine [2]. Standardised creatinine is determined by using a creatinine assay with calibration traceable to an isotope dilution mass spectrometry (IDMS) reference measurement procedure [12], which was developed to enable more accurate renal function estimates and to reduce differences between laboratories. Development of the CKD-EPI equations was based largely on patients with Caucasian or African-American ethnicity and has been evaluated in numerous studies in different patient populations [11]. Of note, the CKD-EPI equation provides a GFR estimate with the unit millilitre/minute per 1.73 m^2^ (eGFR_indexed_), i.e. an estimate normalised to a body surface area (BSA) of 1.73 m^2^, which appears appropriate for detection and staging of CKD [1]. In case of drug dose adjustment, a non-normalised individual estimate with units of millilitre/minute is desired, especially if a patient’s BSA differs significantly from 1.73 m^2^. Thus, in such cases, the estimate should be individualised according to a patient’s BSA (eGFR_non-indexed_ = eGFR_indexed_ / 1.73 · BSA) [1, 11, 13–17]. Interestingly, in a recent analysis, eGFR_non-indexed_ calculated with the CKD-EPI equation performed best at predicting dose requirements for carboplatin, closely followed by eGFR_non-indexed_ calculated with the MDRD equation [18]. In obese patients, eGFR_non-indexed_ calculated with CKD-EPI or MDRD equation was more accurate than eGFR_indexed_ compared with measured ^51^Cr-EDTA or ^99m^Tc-DTPA GFR [19, 20].

The creatinine-based CKD-EPI equation is often implemented in laboratory systems but is also available in online calculators via the Internet. Such freely available online calculators might be used by medical or pharmaceutical professionals when a calculated eGFR is not immediately available from the laboratory or a calculation of eGFR_non-indexed_ is required. The accuracy of such online calculators is unknown.

The aim of the present study was to systematically evaluate the accuracy of freely available online calculators using the creatinine-based CKD-EPI equation.

## Materials and methods

Online calculators using the CKD-EPI equation were identified via Google search using the keywords ‘CKD-EPI’ and ‘CKD-EPI calculator’. Five reference cases were developed, using parameter values that should cover different aspects of the CKD-EPI equation, because actual calculation can be implemented in online calculators as several different equations (where the applicable equation is selected based on sex, ethnicity, and creatinine category) or as a single equation [2]. These hypothetical cases included two Caucasian males with creatinine values below or above 0.9 mg/dl, two Caucasian females with creatinine values below or above 0.7 mg/dl, and one African-American male (Supplementary Table S1). A conversion factor of 88.4 was used to convert creatinine values with a unit of milligramme/decilitre to micromole/litre. BSA was calculated using Mosteller’s equation [21, 22] and Du Bois’ equation [23]. In order to derive the correct eGFR values for the reference cases, the equations (Supplementary Table S2) were entered into two separate Microsoft Excel sheets, which was done independently by two of the authors (SS, DC), and the results compared.

All reference cases were run with each identified online calculator. Deviations from the reference values where an obvious explanation could be identified (e.g. wrong application of BSA, leading to an inverse change in eGFR_non-indexed_) were counted as an error. Deviations without obvious explanation were counted as an error only when the difference to the reference value was ≥ 1 ml/min per 1.73 m^2^. Otherwise, rounding inaccuracies were assumed. If calculators allowed different creatinine units (milligramme/decilitre or micromole/litre), one case was run with both units (in case of an error, the other cases were run with both units also), counting every deviation as an error. Cases where inadequate clinical application would not be expected (e.g. if calculation was not possible because of an upper age limit) were not counted as an error. Analyses were done by a clinical pharmacist (SS) and a clinical pharmacologist and nephrologist (DC).

We checked whether creatinine measurement by a standardised assay and information on the ethnic background of a patient was required and, if not, if the user is informed on these limitations. In addition, information on the online calculators was obtained from the respective site (country of origin, language, type of supplier, and provision of references).

### Statistical analysis

Data was analysed using descriptive statistics. Studied variables are presented with frequency distribution. Calculations were done with the online calculators (using Internet Explorer® 11 (SS) or Firefox Browser® 74.0.1 (DC) on a standard PC with Windows® 10 (SS) or Windows® 7 (DC)) and with Microsoft Excel® 2016 (SS) or 2010 (DC) (Seattle, WA, USA), according to the local standard. Differences between online calculator results when using different versions of the software were not detected.

For cases where errors were identified, the extent of the error was calculated by dividing the online calculator value by the reference value (using the single CKD-EPI equation and Mosteller’s or Du Bois’ equation for BSA, applying the same BSA equation as used by the respective online calculator).

## Results

Overall, 49 online calculators were identified (Supplementary Table S3). Two calculators did not provide any results after entering the values and were not further considered, leading to a total of 47 calculators from 11 countries, mainly Germany (36.2%) and the USA (34.0%) (Table 1). The main languages were English (44.7%) and German (38.3%). The main types of supplier were ‘company-laboratory’ (27.7%), followed by ‘association’ (23.4%) and ‘company-others/unclear’ (21.3%) (Table 1).

**Table 1:** Information on freely available CKD-EPI online calculators

	All tested calculators	Erroneous calculators
Number	47	8
Country of origin		
Germany	17 (36.2)	4 (50.0)
USA	16 (34.0)	
Spain	3 (6.4)	1 (12.5)
France	2 (4.3)	1 (12.5)
Switzerland	2 (4.3)	-
The Netherlands	2 (4.3)	-
Australia	1 (2.1)	1 (12.5)
Canada	1 (2.1)	1 (12.5)
Italy	1 (2.1)	-
Poland	1 (2.1)	-
UK	1 (2.1)	-
Language		
English	21 (44.7)	2 (25.0)
German	18 (38.3)	4 (50.0)
Spanish	3 (6.4)	1 (12.5)
French	2 (4.3)	1 (12.5)
Dutch	1 (2.1)	-
Italian	1 (2.1)	-
Multiple	1 (2.1)	-
Type of supplier		
Company-laboratory	13 (27.7)	2 (25.0)
Association^a^	11 (23.4)	3 (37.5)
Company-others/unclear	10 (21.3)	1 (12.5)
Private person	4 (8.5)	-
Hospital	3 (6.4)	1 (12.5)
Research group	3 (6.4)	-
Company-IT	2 (4.3)	-
Doctor’s practice	1 (2.1)	1 (12.5)

Data are quoted as *n* (%)

^a^Includes medical associations and patient associations

Only 36 of 47 (76.6%) calculators provided accurate estimates for all reference cases (i.e. no errors were detected). After exclusion of two calculators where an arbitrary 70-year age limit prohibited calculation for two cases and one calculator, which did not perform calculations for African-American ethnicity, but explicitly stated that this was the case, 8 (17.0%) calculators were considered as faulty.

Errors concerning ethnicity were found with four calculators. In two cases, no calculation for African-Americans was provided and no information on this fact was given. In one case, the same value was calculated independently from the selected ethnicity. In another case, the calculator provided a wrong eGFR value while an obvious reason could not be identified. For these errors, the average absolute difference from the correct value was 10.9 (range 4.3 to 13.6) ml/min per 1.73 m^2^ with the reference cases. The average eGFR was 91% (range 86 to 104) of the correct value.

Errors concerning individualisation from millilitre/minute per 1.73 m^2^ to millilitre/minute were found with two calculators. Obviously, calculations were done erroneously as eGFR_indexed_ / BSA · 1.73, thus leading to a change in the opposite direction. As only 7 of 47 (14.9%) calculators offered this conversion, 2 of 7 (28.6%) were found to be faulty. For these errors, the average absolute difference was 18.0 (range 0.2 to 49.5) ml/min with the reference cases but could be much larger for patients with very low or very high BSA. The average eGFR was 101% (range 59 to 147) of the correct value.

One calculator produced an eGFR value for one reference case that was consistent with using the CKD-EPI equation for creatinine values > 0.9 mg/dl where application of the equation for values ≤ 0.9 mg/dl would have been correct. The absolute difference was 9.7 ml/min per 1.73 m^2^, and the eGFR was 110%.

An error in conversion of creatinine units between milligramme/decilitre and micromole/litre was found with one calculator. For these errors, the average absolute difference was 2.6 (range 1.4 to 5.2) ml/min per 1.73 m^2^, and the eGFR was 104% (range 101 to 105).

An error in the displayed unit of eGFR was found with one calculator, where millilitre/minute was shown for eGFR_indexed_ and for eGFR_non-indexed_. For these errors, the average absolute difference was 12.0 (range 0.2 to 34.5) ml/min per 1.73 m^2^, and the eGFR was 98% (range 71 and 121) with the reference cases (assuming that eGFR_indexed_ is used where eGFR_non-indexed_ is actually required).

Two calculators produced erroneous eGFR values in one or more reference cases, where no obvious explanation could be identified. For these errors, the average absolute difference was 1.8 (range 1.8 to 2.0) ml/min per 1.73 m^2^, and the eGFR was 102% (range 98 and 102).

The majority of faulty calculators appeared to be German (50.0%). The main type of supplier was ‘association’ (37.5%), followed by ‘company-laboratory’ (25.0%), ‘hospital’ (12.5%), ‘doctor’s practice’ (12.5%), and ‘company-others/unclear’ (12.5%) (Table 1).

In total, there were 28 errors using the 47 calculators and 5 reference cases (1–11 errors per faulty calculator): 8 errors (7 calculators) concerning the calculation of eGFR_indexed_ of which 4 did not have adaption for African-Americans and 4 where eGFR_indexed_-estimates differed more than 1 ml/min per 1.73 m^2^, 10 errors (2 calculators) concerning the conversion to eGFR_non-indexed_, 5 errors (1 calculator) concerning the conversion of creatinine units, and 5 errors (1 calculator) where the units of eGFR_indexed_ were misstated.

**Fig. 1:**
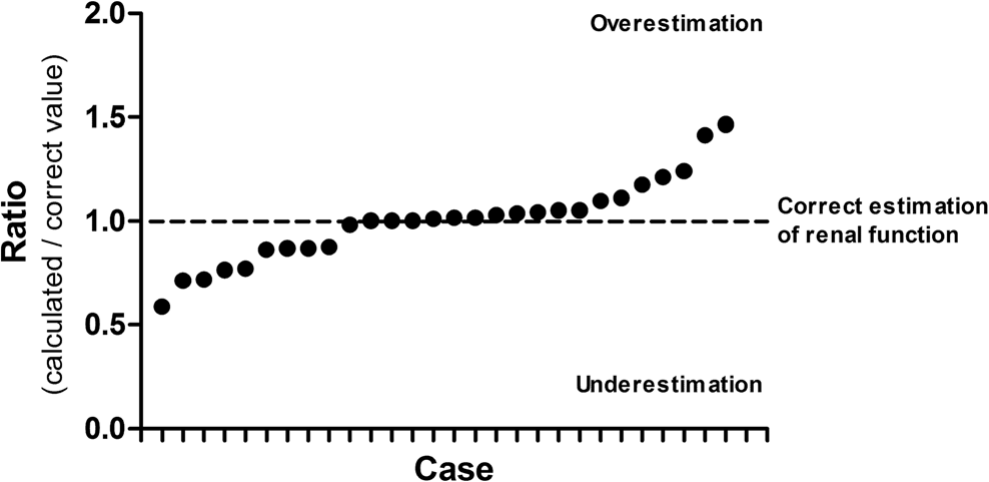
Error in eGFR values calculated by freely available online calculators using the CKD-EPI equation compared with the values of five reference cases (only cases with an obvious explanation or a difference to the reference value of at least 1 ml/min per 1.73 m^2^ are shown)

The extent of these errors ranged between 59 and 147% of the correct value (Fig. 1). When the limit for counting a deviation from the correct value as an error was set at ≥ 2, 5, or 10 ml/min per 1.73 m^2^ (or ml/min), 19, 16, or 11 errors were found, and 7, 6, or 4 calculators were considered as faulty.

Furthermore, 45 (95.7%) of the Internet sites questioned or explicitly referred to ethnicity of a patient, 29 (61.7%) provided references on CKD-EPI, 26 (55.3%) allowed more than one unit of creatinine, 9 (19.1%) explicitly required standardised creatinine values or stated that such values were required, and 5 (10.6%) allowed for calculation of BSA based on height and weight.

## Discussion

Estimation of GFR with the CKD-EPI equation is commonly used for detection and staging of CKD and is recommended for drug dose adjustment of some drugs (after individualisation to eGFR_non-indexed_, where required). After discovering an online calculator with erroneous conversion of units from millilitre/minute per 1.73 m^2^ to millilitre/minute (the index case), we performed a systematic analysis of freely available online calculators using the creatinine-based CKD-EPI equation. From online CKD-EPI calculators, we expect accurate calculations and would like to see a feature to estimate BSA and to calculate the individual eGFR (eGFR_non-indexed_). Since accurate calculations require at least age, sex, ethnicity, and a standardised measurement of serum creatinine, the online calculators should ask for these variables.

Surprisingly, we found that only 36 of 47 (77%) freely accessible online calculators were able to calculate accurate values for our test cases. Eight of 47 (17%) calculators were considered as faulty, because of one or more errors that might lead to inappropriate clinical decision-making, as e.g. in one of the reference cases with erroneous conversion of eGFR_indexed_ of 42.7 ml/min/1.73 m^2^ to eGFR_non-indexed_ of 51.6 ml/min instead of 35.2 ml/min, which could lead to quite different clinical decisions in therapy with metformin. In case of serious adverse events, potential legal implications are easily envisioned, when accurate calculations contraindicate for the use of a drug whereas the erroneous calculation does not. Frequency and consequences of eGFR online calculator use by patients are largely unknown. However, education and self-monitoring of CKD patients are increasingly advocated [24, 25] and information on eGFR is part of patient education material [26]. In a retrospective study, 87% of patients using an electronic health record patient portal reviewed their laboratory results [27]. In a prospective study, providing a specific website to predialysis CKD patients, the GFR calculator was most commonly visited [28]. Thus, increasing eGFR online calculator use by patients can be expected, potentially confusing patients, when erroneous calculators are used. Identified errors included wrong calculations of eGFR for patients with African-American ethnicity, wrong calculation in a case with relatively low creatinine, individualisation of the GFR unit from millilitre/minute per 1.73 m^2^ to millilitre/minute, conversion of the creatinine unit from milligramme/decilitre to micromole/litre, and further errors without obvious explanations.

The extent of the errors ranged from 59 to 147%, which could lead to erroneous staging of CKD and inadequate drug dosing. Even small discrepancies could affect clinical decision-making, if the value is close to the lower limit of a given range (e.g. in case of a drug that is contraindicated in patients with an eGFR below 30 ml/min). Obviously, the extent of the errors identified in the present study depends on the reference cases and could be larger, especially in patients with low or high BMI.

Only 7 of 47 (14.9%) calculators offered the possibility to calculate eGFR_non-indexed_, and in 2 of these, this calculation was incorrect. Calculation eGFR_non-indexed_ may be required for drug dose adjustments for drugs with a narrow therapeutic range (e.g. carboplatin) and in obese patients, where application of eGFR_indexed_ can lead to an erroneously low estimate of renal function and unjustified drug dose adjustment [11, 16, 17, 19, 20]. Two online calculators did not allow calculations for two cases because of age restrictions (> 70 years). Although CKD-EPI might be less accurate in older patients, there appears to be no general recommendation on an upper age limit. Only 9 of 47 (19.1%) of the calculators explicitly asked for creatinine values as measured by a standardised assay. The CKD-EPI equation requires standardised creatinine values, which were used in developing the equation. Non-standardised creatinine values can lead to erroneously low eGFR values [29, 30]. Standardised creatinine can be determined by an enzymatic or alkaline picrate (Jaffé) method with calibration traceable to an IDMS reference [12, 31]. Newer studies recommend the enzymatic method because of an even better performance than the IDMS-traceable Jaffé method [32, 33]. Thus, it might not only be relevant if a standardised creatinine value was used, the method could also be important.

Of note, most providers of online calculators state that their calculators are not to be used for clinical application, are to be used for educational purposes only, or similar. However, such calculators may be used by medical or pharmaceutical professionals and by patients assuming that online calculators are properly evaluated after implementation. The Internet should always be used with caution as a source on information in medicine. However, we expect it will increasingly be used, especially by clinicians with fewer years of experience [34, 35]. To our best knowledge, there are no published reference cases for evaluation of CKD-EPI calculators. Two online calculators from Germany showed the same error in conversion of eGFR units from millilitre/minute per 1.73 m^2^ to millilitre/minute. Thus, it is tempting to speculate that the algorithm from one of these calculators might have been used when programming the other. Thus, the use of reference cases should always be part of software validation.

Our study has some limitations. First, we considered only online calculators that are freely available in the Internet. Thus, our results cannot be generalised to smartphone apps or to calculations implemented in laboratory information systems. Second, background information on a calculator was obtained from the Internet site. In some cases, information, especially on the actual type of provider of the online calculator (e.g. private person or small company), were not entirely clear. However, we refrained from requesting further information from the providers and used what would be available to the typical user.

In conclusion, we found errors in one out of six (17%) freely available CKD-EPI online calculators that might lead to inappropriate clinical decisions if used without critically questioning the results by experienced medical professionals. We strongly recommend that online eGFR calculators (and possibly other calculators also) should be evaluated more thoroughly after implementation and provide reference cases that could be used for creatinine-based CKD-EPI equations. Conversion of normalised eGFR (eGFR_indexed_) to an individual eGFR (eGFR_non-indexed_), which may be needed for drug therapy, should be more often provided.

## Electronic supplementary material

ESM 1 (PDF 443 kb)

## Data Availability

The datasets analysed during the current study are available from the corresponding author on reasonable request.

## References

[1] Kidney Disease: Improving Global Outcomes (KDIGO) CKD Work Group (2013). KDIGO 2012 clinical practice guidelines for the evaluation and management of chronic kidney disease. Kidney Int Suppl.

[2] Levey AS, Stevens LA, Schmid CH, Zhang YL, Castro AF, Feldman HI, Kusek JW, Eggers P, Van Lente F, Greene T (2009). A new equation to estimate glomerular filtration rate. Ann Intern Med.

[3] Cockcroft DW, Gault MH (1976). Prediction of creatinine clearance from serum creatinine. Nephron.

[4] Waller DG, Fleming JS, Ramsey B, Gray J (1991). The accuracy of creatinine clearance with and without urine collection as a measure of glomerular filtration rate. Postgrad Med J.

[5] Park EJ, Wu K, Mi Z, Dong T, Lawrence JP, Ko CW, Huang SM, Zhang L, Crentsil V, Zhang J, Xu NN (2012). A systematic comparison of Cockcroft-Gault and modification of diet in renal disease equations for classification of kidney dysfunction and dosage adjustment. Ann Pharmacother.

[6] Hellden A, Bergman U, Odar-Cederlof I (2019). The importance of correct estimation of renal function for drug treatment in hospitalized elderly patients, especially women: a prospective observational study. Clin Nephrol.

[7] Dowling TC, Wang ES, Ferrucci L, Sorkin JD (2013). Glomerular filtration rate equations overestimate creatinine clearance in older individuals enrolled in the Baltimore Longitudinal Study on Aging: impact on renal drug dosing. Pharmacotherapy.

[8] Cartet-Farnier E, Goutelle-Audibert L, Maire P, De la Gastine B, Goutelle S (2017). Implications of using the MDRD or CKD-EPI equation instead of the Cockcroft-Gault equation for estimating renal function and drug dosage adjustment in elderly patients. Fundamental Clin Pharmacol.

[9] Schwartz JB (2016). Potential effect of substituting estimated glomerular filtration rate for estimated creatinine clearance for dosing of direct oral anticoagulants. J Am Geriatr Soc.

[10] Andrade JG, Hawkins NM, Fordyce CB, Deyell MW, Er L, Djurdjev O, Macle L, Virani SA, Levin A (2018). Variability in non-vitamin K antagonist oral anticoagulants dose adjustment in atrial fibrillation patients with renal dysfunction: the influence of renal function estimation formulae. Can J Cardiol.

[11] Levey AS, Inker LA (2017). Assessment of glomerular filtration rate in health and disease: a state of the art review. Clin Pharmacol Ther.

[12] National Institute of Diabetes and Digestive and Kidney Disease: creatinine standardization recommendations, online https://www.niddk.nih.gov/health-information/professionals/clinical-tools-patient-management/kidney-disease/laboratory-evaluation/glomerular-filtration-rate/creatinine-standardization/recommendations. 10.04.2020

[13] Munar MY, Singh H (2007). Drug dosing adjustments in patients with chronic kidney disease. Am Fam Physician.

[14] National Kidney Foundation (2014). Frequently asked questions about GFR estimates.

[15] European Medicines Agency (2015) Guideline on the evaluation of the pharmacokinetics of medicinal products in patients with decreased renal function. https://www.ema.europa.eu/en/documents/scientific-guideline/guideline-evaluation-pharmacokinetics-medicinal-products-patients-decreased-renal-function_en.pdf

[16] Stevens LA, Nolin TD, Richardson MM, Feldman HI, Lewis JB, Rodby R, Townsend R, Okparavero A, Zhang YL, Schmid CH, Levey AS (2009). Comparison of drug dosing recommendations based on measured GFR and kidney function estimating equations. American journal of kidney diseases : the official journal of the National Kidney Foundation.

[17] Czock D, Bertsche T, Haefeli WE (2009). Drug dose adjustments in patients with renal impairment. American journal of kidney diseases : the official journal of the National Kidney Foundation.

[18] Janowitz T, Williams EH, Marshall A, Ainsworth N, Thomas PB, Sammut SJ, Shepherd S, White J, Mark PB, Lynch AG, Jodrell DI, Tavare S, Earl H (2017). New model for estimating glomerular filtration rate in patients with cancer. Journal of clinical oncology : official journal of the American Society of Clinical Oncology.

[19] Bouquegneau A, Vidal-Petiot E, Moranne O, Mariat C, Boffa JJ, Vrtovsnik F, Scheen AJ, Krzesinski JM, Flamant M, Delanaye P (2016). Creatinine-based equations for the adjustment of drug dosage in an obese population. Br J Clin Pharmacol.

[20] Chew-Harris JS, Chin PK, Florkowski CM, George P, Endre Z (2015). Removal of body surface area normalisation improves raw-measured glomerular filtration rate estimation by the Chronic Kidney Disease Epidemiology Collaboration equation and drug dosing in the obese. Intern Med J.

[21] Mosteller RD (1987). Simplified calculation of body-surface area. N Engl J Med.

[22] Lam TK, Leung DT (1988). More on simplified calculation of body-surface area. N Engl J Med.

[23] Du Bois D, Du Bois EF (1989). A formula to estimate the approximate surface area if height and weight be known. 1916. Nutrition (Burbank, Los Angeles County, Calif).

[24] Oliveira JGR, Askari M, Silva Junior GBD, Freitas Filho RA, Vasconcelos Filho JE (2019). Renal health: an innovative application to increase adherence to treatment through self-monitoring for patients with CKD and provide information for the general population. Kidney international reports.

[25] Narva AS, Norton JM, Boulware LE (2016). Educating patients about CKD: the path to self-management and patient-centered care. Clinical journal of the American Society of Nephrology : CJASN.

[26] National Kidney Foundation. Estimated glomerular filtration rate (eGFR), online https://www.kidney.org/atoz/content/gfr. 26.04.2020

[27] Jhamb M, Cavanaugh KL, Bian A, Chen G, Ikizler TA, Unruh ML, Abdel-Kader K (2015). Disparities in electronic health record patient portal use in nephrology clinics. Clinical journal of the American Society of Nephrology : CJASN.

[28] Diamantidis CJ, Fink W, Yang S, Zuckerman MR, Ginsberg J, Hu P, Xiao Y, Fink JC (2013). Directed use of the internet for health information by patients with chronic kidney disease: prospective cohort study. J Med Internet Res.

[29] Stevens LA, Levey AS (2009). Use of the MDRD study equation to estimate kidney function for drug dosing. Clin Pharmacol Ther.

[30] Murthy K, Stevens LA, Stark PC, Levey AS (2005). Variation in the serum creatinine assay calibration: a practical application to glomerular filtration rate estimation. Kidney Int.

[31] Myers GL, Miller WG, Coresh J, Fleming J, Greenberg N, Greene T, Hostetter T, Levey AS, Panteghini M, Welch M, Eckfeldt JH (2006). Recommendations for improving serum creatinine measurement: a report from the Laboratory Working Group of the National Kidney Disease Education Program. Clin Chem.

[32] Piéroni L, Bargnoux AS, Cristol JP, Cavalier E, Delanaye P (2017). Did creatinine standardization give benefits to the evaluation of glomerular filtration rate?. Ejifcc.

[33] Jassam N, Weykamp C, Thomas A, Secchiero S, Sciacovelli L, Plebani M, Thelen M, Cobbaert C, Perich C, Ricos C, Paula FA, Barth JH (2017). Post-standardization of routine creatinine assays: are they suitable for clinical applications. Ann Clin Biochem.

[34] Dziadzko MA, Gajic O, Pickering BW, Herasevich V (2016). Clinical calculators in hospital medicine: availability, classification, and needs. Comput Methods Prog Biomed.

[35] Green TA, Whitt S, Belden JL, Erdelez S, Shyu CR (2019). Medical calculators: prevalence, and barriers to use. Comput Methods Prog Biomed.

