# Supplementary material for: Accuracy of freely available online GFR calculators using the CKD-EPI equation
Source: Eur J Clin Pharmacol. 2020 Jun 19;76(10):1465–70. doi: 10.1007/s00228-020-02932-x (PMC7481157; doi:10.1007/s00228-020-02932-x)
Supplement: Supplementary file 1 — (PDF 443 kb) [file 228_2020_2932_MOESM1_ESM.pdf]

European Journal of Clinical Pharmacology

Accuracy of freely available online GFR calculators using the CKD-EPI equation

Sarah Seiberth, Theresa Terstegen, Dorothea Strobach, David Czock*

Table S1: Reference cases.

	Case 1	Case 2	Case 3	Case 4	Case 5
Age [years]	80	45	58	78	22
Sex	Female	Female	Male	Male	Male
Ethnicity	Caucasian	Caucasian	Caucasian	Caucasian	Afro-American
Creatinine [mg/dl]	1.20	0.60	0.80	1.80	1.20
Creatinine [µmol/l]	106.08	53.04	70.72	159.12	106.08
Height [cm]	163.00	173.00	178.00	174.00	170.00
Weight [kg]	45.00	62.00	79.00	50.00	93.00
$eGFR_{indexed}^{(1)}$	42.65	110.08	98.47	35.26	98.89
$eGFR_{indexed}^{(2)}$ [ml/min per 1.73 m^2]	42.79	110.43	98.47	35.26	98.63

	Case 1		Case 2		Case 3		Case 4		Case 5	
BSA equation	**M**	**D**	**M**	**D**	**M**	**D**	**M**	**D**	**M**	**D**
BSA [m^2]	1.43	1.45	1.73	1.74	1.98	1.97	1.55	1.60	2.10	2.04
$eGFR_{non\text{-}indexed}^{(1)}$	35.19	35.87	109.83	110.76	112.49	112.12	31.69	32.52	119.79	116.72
$eGFR_{non\text{-}indexed}^{(2)}$ [ml/min]	35.30	35.98	110.19	111.12	112.49	112.12	31.69	32.52	119.48	116.42

D: Du Bois' equation. **M:** Mosteller's equation.
(1) Single, general CKD-EPI equation [Levey 2009].
(2) Specific CKD-EPI equations for the four categories, using rounded values as presented by Levey et al. [Levey 2009].

Levey AS, Stevens LA, Schmid CH, et al (2009) A new equation to estimate glomerular filtration rate. Ann Intern Med 150:604-612

* Corresponding author: David Czock, University Hospital Heidelberg, david.czock@med.uni-heidelberg.de

Table S2: Equations for calculation of $eGFR_{indexed}$ (using CKD-EPI equations) and $eGFR_{non\text{-}indexed}$ (using body surface area, BSA).

$eGFR_{indexed}$
**Caucasian female and creatinine ≤ 0.7 mg/dl:**
$eGFR\ [ml/min/1.73\ m^2] = 143.538\ \cdot 0.993^{age} \cdot (\frac{standard.creatinine}{0.7})^{-0.329}$
**Caucasian female and creatinine > 0,7 mg/dl:**
$eGFR\ [ml/min/1.73\ m^2] = 143.538\ \cdot 0.993^{age} \cdot (\frac{standard.creatinine}{0.7})^{-1.209}$
**Caucasian male and creatinine ≤ 0,9 mg/dl:**
$eGFR\ [ml/min/1.73\ m^2] = 141 \cdot 0.993^{age} \cdot (\frac{standard.creatinine}{0.9})^{-0.411}$
**Caucasian male and creatinine > 0,9 mg/dl:**
$eGFR\ [ml/min/1.73\ m^2] = 141 \cdot 0.993^{age} \cdot (\frac{standard.creatinine}{0.9})^{-1.209}$
**For patients with African-American ethnicity multiply by 1.159.**
**BSA**
**Mosteller's equation**
BSA [m^2] = (height [cm] · weight [kg] / 3600)$^{0.5}$
**Du Bois' equation**
BSA [m^2] = 0.007184 · height [cm]$^{0.725}$ x weight [kg]$^{0.425}$
**$eGFR_{non\text{-}indexed}$**
$eGFR_{non-indexed}\ [ml/min] = \frac{eGFR_{indexed}\ [ml/min/1.73\ m^2]}{1.73} \cdot BSA\ [m^2]$

Table S3: CKD-EPI online calculators (n = 49)

www.arztpraxistreusch.de/onlinedienstegfr.php
www.bioscientia.de/de/service/medizinische-formeln/gfr-nach-ckdepi-formel/
www.blackholm.com/service/rechentools/gfr-epi-formel/
www.calculator.net/gfr-calculator.htm
www.cbm25.fr/formule-creatinine
www.ckdepi.org/equations/gfr-calculator/
www.clincalc.com/Kinetics/CrCl.aspx
www.columbiamedicine.org/divisions/gharavi/calc_egfr.php
www.davita.com/tools/gfr-calculator
www.ebmconsult.com/app/medical-calculators/glomerular-filtration-rate-gfr-calculator
www.evidencio.com/models/show/413
www.globalrph.com/medcalcs/chronic-kidney-disease-epidemiology-collaboration-ckd-epi-calculator/
www.hdcn.com/calc.htm
www.kidney.org.au/health-professionals/detect/calculator-and-tools
www.kidney.org/professionals/KDOQI/gfr_calculator
www.knmp.nl/rekenmodules/creatinine_html
www.kreatinin-clearance-rechner.ch/
www.lab-kl.de/aerzte/rechenprogramme/gfr-n-ckdepi/
www.labopart.de/einsender/formelberechnungen/glomerulaere-filtration/
www.labor-clotten.de/GFR-nach-CKD-EPI-For.544.0.html
www.labor-dortmund.de/index.php?id=90
www.laborkrone.de/testsite/en/medical-practitioners/calculator/
www.labor-limbach.de/laborrechner/labor-rechner/gfr-nach-ckd-epi-formel/
www.ladr.de/service/rechenprogramme/gfr-ckd
www.mdapp.co/egfr-calculator-by-ckd-epi-79/
www.mdcalc.com/ckd-epi-equations-glomerular-filtration-rate-gfr
www.merckmanuals.com/medical-calculators/GFR_CKD_EPI.htm
www.mqzh.ch/cm/en/egfr-e.html
www.msdmanuals.com/en-kr/professional/multimedia/clinical-calculator/glomerular%20filtration%20rate%20estimate%20by%20ckd%20epi%20equation
www.mvz-labor-lb.de/diagnostik/laborrechner/formelrechner.html
www.nephrologisch.de/tools.html
www.nephromatic.com/egfr.php
www.niddk.nih.gov/health-information/communication-programs/nkdep/laboratory-evaluation/glomerular-filtration-rate-calculators/ckd-epi-adults-si-units
www.nierenrechner.de/index.php?page=egfr-ckd-rechner
www.omnicalculator.com/health/glomerular-filtration-rate
www.qxmd.com/calculate/calculator_251/egfr-using-ckd-epi
www.samiuc.es/calculo-del-filtrado-glomerular-ckd-epi/
www.schenk-ansorge.de/labor/rechner/gfr-ckd-epi/211
www.scymed.com/en/smnxps/psdgt313.htm
www.semergencantabria.org/calc/cucalc2.htm
www.senefro.org/modules.php?name=calcfg
www.sfndt.org/sn/eservice/calcul/eDFG.htm
www.siditalia.it/clinica/formule-e-calcolatori/gfr-stimato-con-epi-ckd
tavarelab.cruk.cam.ac.uk/JanowitzWilliamsGFR/
www.touchcalc.com/calculators/epi
www.ukidney.com/sodium-case-simulations/61-special-presentations/1415-egfr-and-ckd-epi
www.uniklinik-ulm.de/zentrale-einrichtung-klinische-chemie/berechnungen.html
www.uniklinikum-saarland.de/einrichtungen/kliniken_institute/zentrallabor/formeln_und_scores/gfr_kalkulator/
www.wisplinghoff.de/fuer-aerzte/formelsammlung/niere-gfr-nach-ckd-epi-formel-chronic-kidney-disease-epidemiology-collaboration/
